# Aryl hydrocarbon receptor (AhR)-dependent regulation of pulmonary miRNA by chronic cigarette smoke exposure

**DOI:** 10.1038/srep40539

**Published:** 2017-01-12

**Authors:** Sarah Rogers, Angela Rico de Souza, Michela Zago, Matthew Iu, Necola Guerrina, Alvin Gomez, Jason Matthews, Carolyn J. Baglole

**Affiliations:** 1 Departments of Medicine, McGill University, Montreal, Quebec, Canada; 2 Research Institute of the McGill University Health Centre (RI-MUHC), Meakins-Christie Laboratories, Montreal, QC, Canada; 3 Departments of Pharmacology & Therapeutics, McGill University, Montreal, Quebec, Canada; 4 Departments of Pathology, McGill University, Montreal, Quebec, Canada; 5 Department of Pharmacology & Toxicology, University of Toronto, Toronto, Ontario, Canada; 6 Department of Nutrition, University of Oslo, Oslo, Norway

## Abstract

The aryl hydrocarbon receptor (AhR) is a ligand-activated transcription factor historically known for its toxic responses to man-made pollutants such as dioxin. More recently, the AhR has emerged as a suppressor of inflammation, oxidative stress and apoptosis from cigarette smoke by mechanisms that may involve the regulation of microRNA. However, little is known about the AhR regulation of miRNA expression in the lung in response to inhaled toxicants. Therefore, we exposed *Ahr*^−/−^ and *Ahr*^+/−^ mice to cigarette smoke for 4 weeks and evaluated lung miRNA expression by PCR array. There was a dramatic regulation of lung miRNA by the AhR in the absence of exogenous ligand. In response to cigarette smoke, there were more up-regulated miRNA in *Ahr*^−/−^ mice compared to *Ahr*^+/−^ mice, including the cancer-associated miRNA miR-96. There was no significant change in the expression of the AhR regulated proteins HuR and cyclooxygenase-2 (COX-2). There were significant increases in the anti-oxidant gene sulfiredoxin 1 (Srxn1) and FOXO3a- predicted targets of miR-96. Collectively, these data support a prominent role for the AhR in regulating lung miRNA expression. Further studies to elucidate a role for these miRNA may further uncover novel biological function for the AhR in respiratory health and disease.

The aryl hydrocarbon receptor (AhR) is a member of the basic helix-loop-helix Per-Arnt-Sim (bHLH-PAS) transcription factor family that is well-known to mediate the toxicological responses of environmental contaminants such as 2,3,7,8-tetrachlorodibenzo-*p*-dioxin (TCDD). Other ligands for the AhR include polycyclic aromatic hydrocarbons (PAHs) such as benzo[*a*]pyrene (B[*a*]P), a component of ambient air pollution and cigarette smoke. In the absence of ligand, the AhR is found in the cytoplasm complexed with chaperone proteins, including a dimer of heat shock protein 90 (HSP90) and the immunophilin hepatitis B virus X-associated protein 2 (XAP2)^1^,^2^,^3^. After binding ligand, the AhR translocates to the nucleus, dissociates from these chaperones and forms a heterodimer with the AhR nuclear transporter (ARNT). This AhR:ARNT complex then binds to a dioxin responsive element (DRE; also called xenobiotic response element (XRE) or AhR response element (AhRE)) and initiates the transcription of genes that comprise the AhR gene battery, the prototypical of which are the Phase I cytochrome P450 (CYP) enzymes such as CYP1A1.

While prolonged activation of this AhR pathway by dioxin is typically associated with toxic responses (*e*.*g*. cleft palate, hepatomegaly), a broad range of biochemical and genetic studies have now demonstrated that the AhR is essential for many biological functions, including liver development, the induction of endotoxin tolerance and resistance to infection^4^,^5^,^6^,^7^. Our published data show that the AhR is a potent suppressor of inflammation, oxidative stress and apoptosis caused by exposure to cigarette smoke, the leading cause of preventable death worldwide^8^,^9^,^10^,^11^,^12^,^13^. Many of the protective functions of the AhR against the deleterious effects of cigarette smoke occurred by a mechanism that is independent of classic DRE binding. The mechanism by which the AhR suppresses inflammatory and cell death pathways is unclear but we hypothesize that it involves AhR-dependent regulation of microRNA (miRNA), single-stranded, non-coding, 22 nucleotide-long RNA which act posttranscriptionally to inhibit protein expression^14^,^15^. More than 1000 miRNA exist in humans, and it is estimated that ≈30% of the human genome is regulated by miRNA^16^. Mature miRNAs guide the miRNA-induced silencing complex (miRISC) to the 3′ untranslated region of an mRNA strand which the miRNA can bind to with complementarity. If the miRNA binds to the mRNA with close-to-perfect pairing, the miRISC cleaves the mRNA, causing its degradation^17^. Alternatively, if the miRNA binds the mRNA with less complementarity, the miRISC can inhibit the translation of the transcript; both of these result in inhibition of protein production^18^. Since the elucidation of the role of the miRNA lin-14 and lin-4 in the developmental timing of *C. Elegans*, miRNA have been a burgeoning topic of research. While the transcription factor(s) that control miRNA expression in response to smoke are less well-described, we have shown that the AhR controls the basal expression of miR-196a in primary lung fibroblasts^12^. Whether the AhR exerts control over pulmonary miRNA expression in response to cigarette smoke is not known. Therefore, we utilized a chronic *in vivo* cigarette smoke exposure model to evaluate the differential regulation of pulmonary miRNA levels in *Ahr*^+/−^ and *Ahr*^−/−^ mice. Our data show that the AhR is involved in the selective modulation of miRNA expression by cigarette smoke, and in particular suppressing levels of miR-96, a miRNA strongly implicated in cancer progression. The AhR also suppresses pulmonary inflammation in response to chronic smoke exposure. A predicted target of miR-96 is Forkhead box O3 (FOXO3a), a transcription factor that negatively regulates inflammation and oxidative stress^19^,^20^,^21^. We now show for the first time that the AhR increased the expression of FOXO3a in response to cigarette smoke. When considered as a whole, the suppression of miR-96 may increase expression of FOXO3a and be how the AhR attenuates inflammation in response to cigarette smoke. These data shed light on a novel role for the AhR in the regulation of pulmonary miRNA expression and hint towards endogenous effector functions for the AhR in maintaining respiratory health.

## Results

### *Ahr* ^−/−^ mice exhibit enhanced pulmonary neutrophilia in response to chronic cigarette smoke exposure that is not due to increased levels of chemotactic cytokines

**Figure 1:**
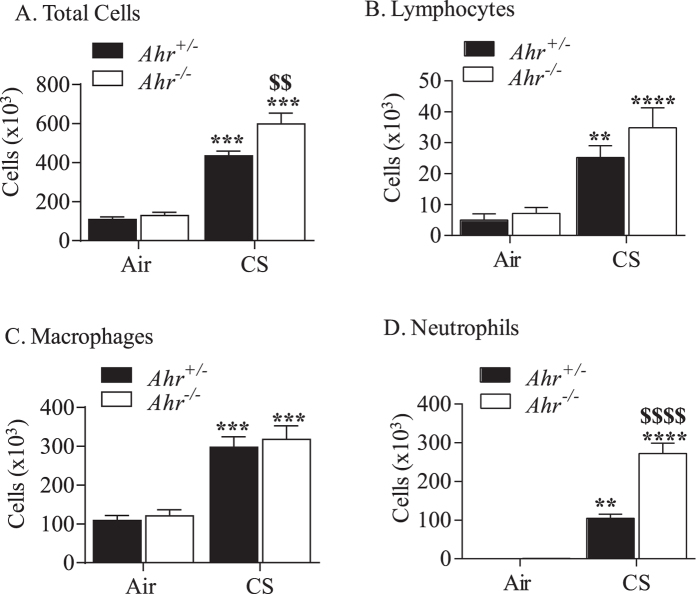
Elevated pulmonary neutrophilia in *Ahr*^−/−^ mice exposed to cigarette smoke for 4 weeks. *Ahr*-expressing mice (*Ahr*^+/−^, *black bars*) and *Ahr*^−/−^ mice (*white bars*) were exposed to cigarette smoke or room air for 4 weeks, sacrificed after the last exposure and differential cell counts performed on the BAL. (**A**) Total Cells- there was a significant increase in total BAL cell numbers in *Ahr*^−/−^ mice exposed to cigarette smoke (CS) compared to air (***p < 0.001) as well as compared to smoke-exposed *Ahr*^+/−^ mice (^$$^p < 0.01). (**B**) Lymphocytes- there was a significant elevation in the number of BAL lymphocytes in *Ahr*^+/−^ and *Ahr*^−/−^ mice exposed to CS (**p < 0.01; ***p < 0.0001). (**C**) Macrophages- the number of macrophages in the BAL was significantly higher after 4 weeks of cigarette smoke exposure (***p < 0.0001 compared to respective air controls) (**D**) Neutrophils- there was a significant increase in the number of neutrophils in the BAL of *Ahr*^−/−^ mice exposed to CS compared to both *Ahr*^−/−^ mice exposed to air (****p < 0.00001) and *Ahr*^+/−^ mice exposed to CS (^$$$$^p < 0.00001). Results are presented as the mean ± SEM (n = 4–5 mice per group).

We have previously published that the AhR suppresses acute and sub-chronic cigarette smoke-induced pulmonary inflammation, including neutrophil influx to the lung^10^,^22^. Whether the AhR is capable of suppressing neutrophilia in response to prolonged exposure is not known. To address this, we exposed *Ahr*^+/−^ and *Ahr*^−/−^ mice to cigarette smoke daily for 4 weeks. This exposure regime significantly increased total number of BAL cells in the *Ahr*^−/−^ mice in response to smoke compared to both air-exposed *Ahr*^−/−^ mice as well as smoke-exposed *Ahr*^+/−^ mice (Fig. 1A). There was a significant increase in lymphocytes and macrophages in cigarette smoke-exposed mice, but there was no difference between *Ahr*^−/−^ and *Ahr*^+/−^ mice (Fig. 1B and C). Exposure of *Ahr*^−/−^ mice to cigarette smoke however significantly increased the number of lung neutrophils compared to smoke-exposed *Ahr*^+/−^ mice (Fig. 1D), supporting that the AhR maintains protection in the lung against excessive neutrophilic inflammation.

**Figure 2:**
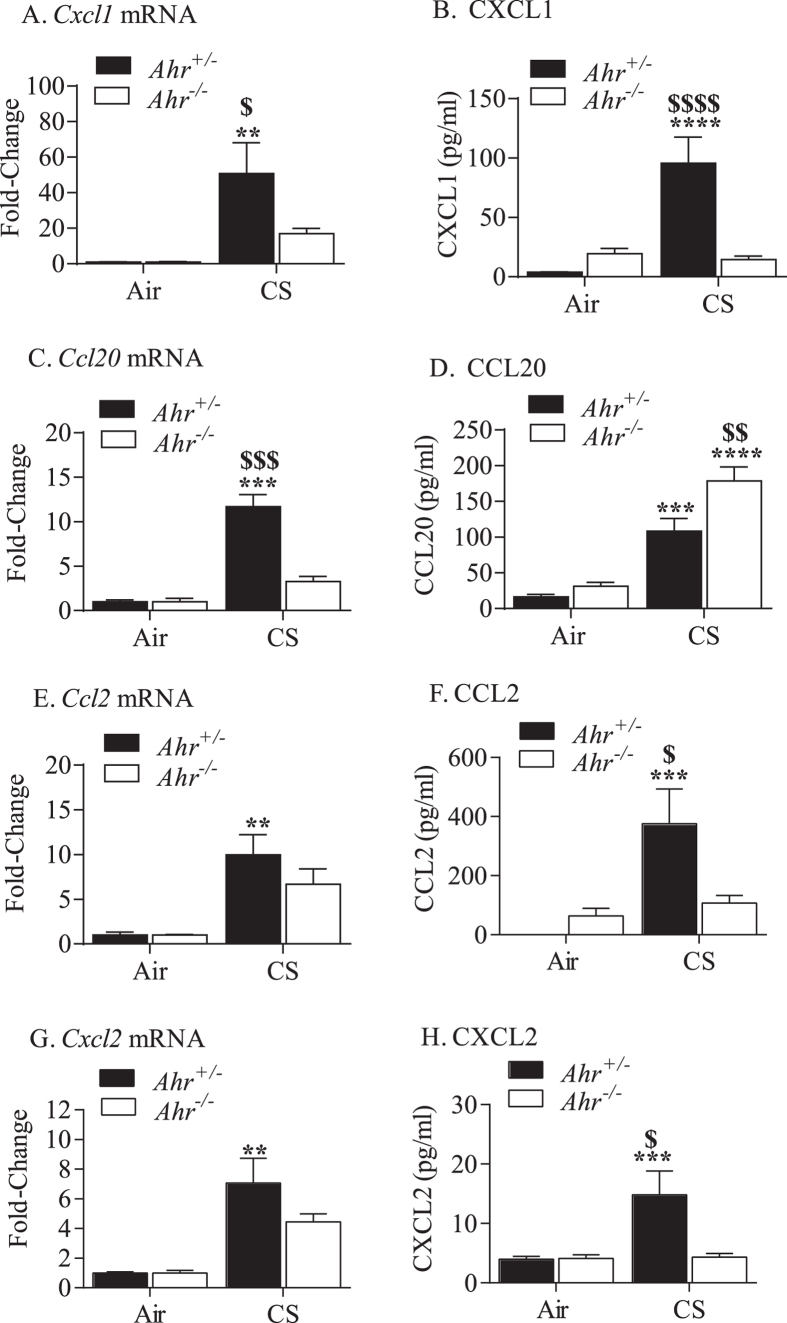
Cytokine expression in cigarette smoke-exposed *Ahr*^−/−^ and *Ahr*^+/−^ mice. *Ahr*^−/−^ and *Ahr*^+/−^ mice were exposed to cigarette smoke for 4 weeks and whole lung homogenates processed for qRT-PCR; the BAL was also collected and cell-free supernatant analyzed for cytokine expression using multiplex array. Cytokines analyzed included (mRNA, protein) (**A**,**B**) CXCL1, (**C**,**D**) CCL20, (**E**,**F**) CCL2 and (**G**,**H**) CXLC2 (**p < 0.01,***p < 0.001 and ****p < 0.00001 compared with respective control; ^$^p < 0.05, ^$$^p < 0.01, ^$$$^p < 0.001, and ^$$$$^p < 0.00001 between smoke exposed *Ahr*^−/−^ and *Ahr*^+/−^ mice). Results are presented as the mean ± SEM (n = 3–5 mice per group); mRNA values were normalized to housekeeping (*β-actin* or GAPDH).

Neutrophil recruitment to the lung during injury or infection follows a cascade of tethering, rolling, adhesion, crawling, and transmigration, events that are mediated by chemokines and cytokines. Control over the levels of chemotactic cytokines occurs at both the transcriptional and posttranscriptional levels, the latter also being regulated by miRNA. As the AhR suppression of pulmonary neutrophilia in response to chronic smoke exposure may involve regulation at both of these levels, we examined mRNA and protein expression of key cytokines, including CXCL1 (Gro-α/KC) (Fig. 2A and B), CCL20 (macrophage inflammatory protein-3α [MIP-3α]) (Fig. 2C and D), CCL2 (monocyte chemotactic protein-1 [MCP-1]) (Fig. 2E and F) and CXCL2 (macrophage inflammatory protein-2α [MIP-2α]) (Fig. 2G and H). As there was generally less induction of these cytokines in smoke-exposed *Ahr*^−/−^ mice (Fig. 2), it is unlikely that differential levels of chemotactic cytokines can account for the significant increase in pulmonary neutrophilia in *Ahr*^−/−^ mice in response to chronic cigarette smoke exposure.

### Genetic ablation of the AhR causes dysregulation of basal pulmonary miRNA expression

**Figure 3:**
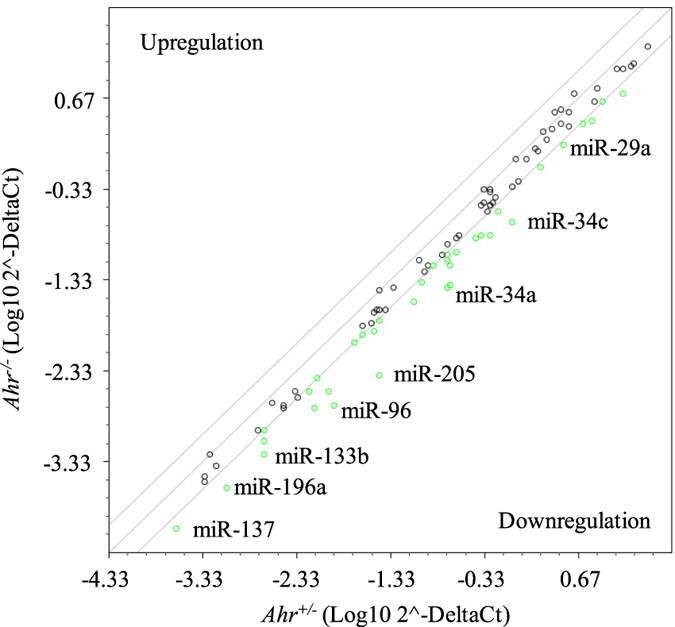
Genetic ablation of the AhR causes alterations in basal expression of pulmonary miRNA expression. miRNA expression in the lungs of naive *Ahr*^−/−^ and *Ahr*^+/−^ mice was analyzed by a RT^2^ miRNA qPCR array. One representative sample per exposure condition/AhR genotype was randomly chosen on which to perform the array. Equivalent miRNA expression is represented by the central diagonal line, whereas the outer diagonal lines represent 2-fold changes in miRNA expression between *Ahr*^−/−^ and *Ahr*^+/−^ mice. Circles in green are miRNAs whose expression was 2-fold lower in *Ahr*^−/−^ mice compared to *Ahr*^+/−^ mice.

To better understand how the AhR might offer protection in the lung, we evaluated miRNA expression. miRNA are key regulators of protein expression by governing mRNA stability and/or translation repression. Given that we have shown there is AhR-dependent regulation of miR-196a in lung fibroblasts^12^, and that the AhR can control mRNA stability of *Cox-2* in response to cigarette smoke^9^, we evaluated whether the presence of the AhR affects pulmonary miRNA levels in response to chronic smoke exposure model using a commercial miRNA array that compares approximately 84 miRNAs. Our cigarette smoke exposure regime is highly relevant to human exposures, as people often smoke for many years/decades^22^. First we evaluated whether there were any basal differences in pulmonary miRNA in the lung of naïve mice. A number of miRNAs were identified to have over two-fold differences in expression in air-exposed *Ahr*^−/−^ compared to the *Ahr*^+/−^ mice. These miRNA included miR-196a, miR-96 and miR-34c (Fig. 3, *green circles*). These miRNAs represent those that are regulated by the AhR in the absence of exogenous ligand.

### Chronic cigarette smoke differentially regulates miRNA levels in an AhR-dependent manner

**Figure 4:**
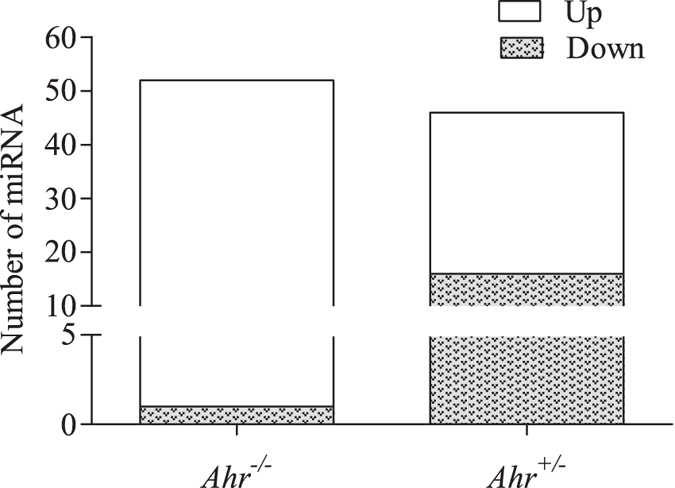
Comparison of miRNA regulation between cigarette smoke-exposed *Ahr*^−/−^ and *Ahr*^+/−^ mice. miRNA analysis after 4 weeks of exposure to cigarette smoke indicated that there were approximately 62 miRNA altered by smoke exposure. *Ahr*^−/−^ mice had more miRNA that were upregulated compared to *Ahr*^+/−^ mice. *Ahr*^+/−^ mice had more pulmonary miRNA that were down-regulated by chronic smoke exposure.

**Figure 5:**
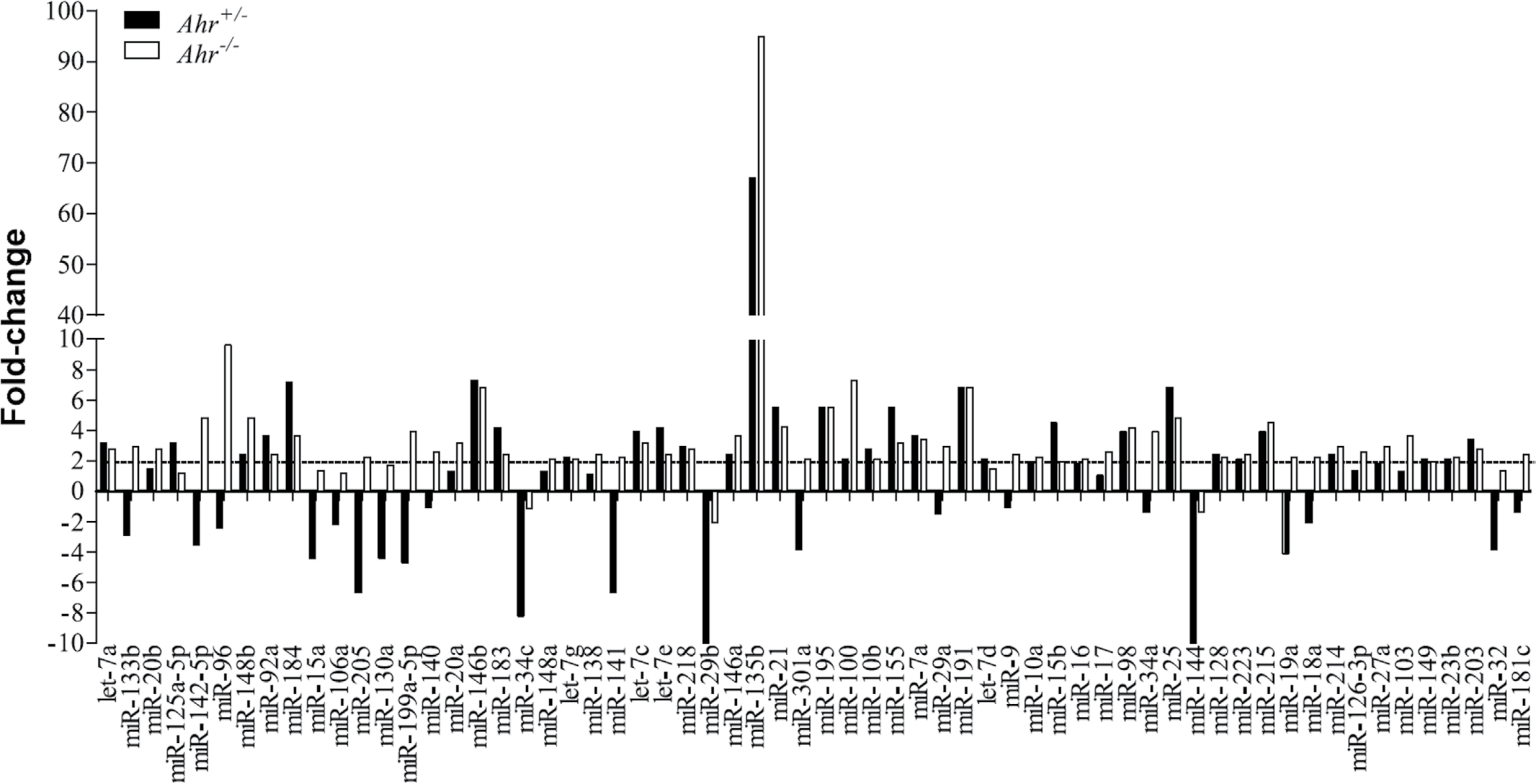
Regulation of cigarette smoke-induced pulmonary miRNA by the AhR. miRNA analysis after 4 weeks of exposure to cigarette smoke was evaluated by PCR array in the lungs of *Ahr*^−/−^ and *Ahr*^+/−^ mice. Threshold was set at 2-fold. There was a dramatic upregulation of miR-135b and a more modest change in miR-146a whereas miR-96 was only increased in cigarette smoke-exposed *Ahr*^−/−^ mice. Values are normalized to housekeeping comparisons made to the respective air-only control mice.

**Figure 6:**
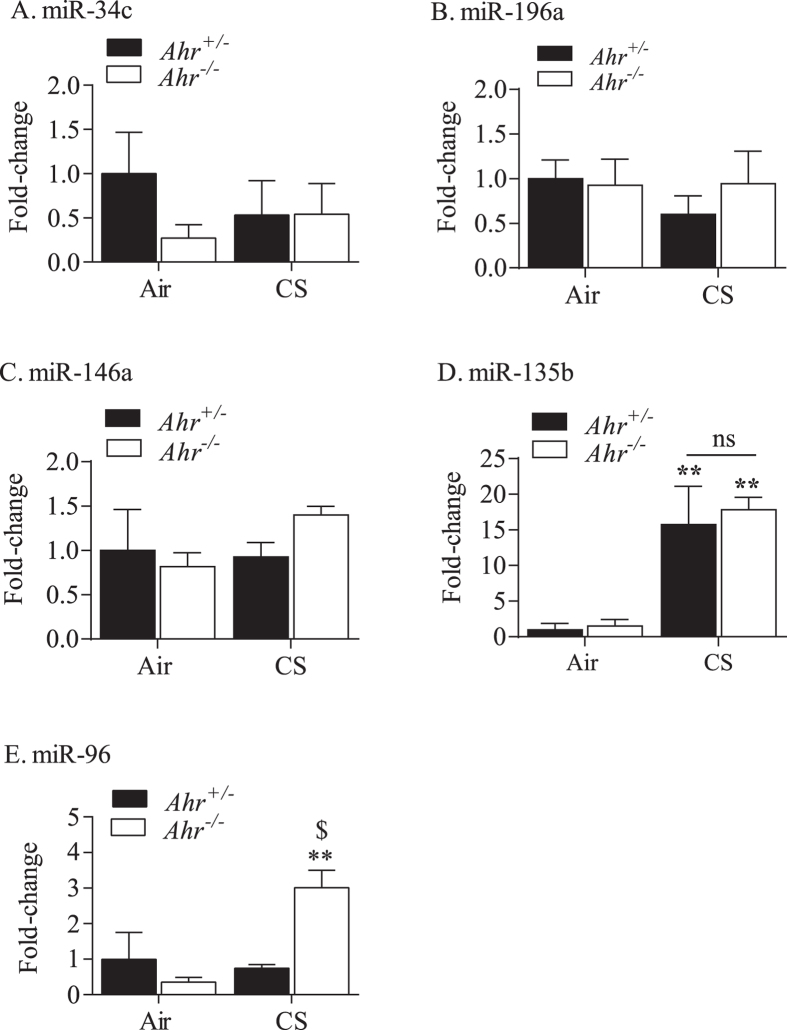
Induction of miR-96 by cigarette smoke is regulated by the AhR. miRNA validation after 4 weeks of exposure to cigarette smoke by qRT-PCR in the lungs of *Ahr*^−/−^ and *Ahr*^+/−^ mice. There was no change in the expression of (**A**) miR-34c, (**B**) miR-196a or (**C**) miR-146a in response to cigarette smoke. There was a significant induction in miR-135b (**D**) in both *Ahr*^−/−^ and *Ahr*^+/−^ mice after smoke (**p < 0.01 compared to respective air control). There is significant difference in miR-96 expression between *Ahr*^−/−^ mice exposed to cigarette smoke (**E**) (**p < 0.01 compared to air control). There was also a significant difference in miR-96 expression between smoke exposed *Ahr*^−/−^ and *Ahr*^+/−^ mice (^$^p < 0.05). Results are presented as the mean ± SEM (n = 4–5 mice per group). Statistical analysis was performed by a two-way ANOVA followed by a Bonferroni’s post hoc test.

We next compared whether there was differential regulation of miRNAs after cigarette smoke exposure for 4 weeks. Preliminary analysis revealed that there were more up-regulated miRNA in the lungs of *Ahr*^−/−^ compared to that of *Ahr*^+/−^ mice after exposure to smoke (Fig. 4). In contrast, *Ahr*^+/−^ mice had more miRNAs that were down-regulated by chronic smoke exposure (Fig. 4). Overall, approximately 62 miRNAs exhibited at least a two-fold difference in relative expression after cigarette smoke exposure (Fig. 4). Several of the miRNAs exhibiting a slight increase are those previously associated with cigarette smoke-induced inflammation, including miR-146a^23^,^24^ (approximately 2-fold; Fig. 5). The miRNA with the largest fold-change was miR-135b (approximately 71-fold in *Ahr*^+/−^ mice) (Fig. 5). Therefore, we next selected miR-146a and miR-135b in conjunction with miRNA exhibiting large relative differences between *Ahr*^−/−^ and *Ahr*^+/−^ mice; these were miR-96 and miR-34C. We also selected for validation by qRT-PCR miRNA that were not altered by smoke exposure, including miR-196a (data not shown). These analyses revealed that there were no significant change in the expression of miR-34c (Fig. 6A), miR-196a (Fig. 6B) or miR-146a (Fig. 6C) in mice exposed to cigarette smoke for 4 weeks. There was also no significant difference in the relative levels of these three miRNA based on AhR expression, although there was a trend towards decreased miR-34c. Cigarette smoke significantly increased miR-135b in the lungs of both *Ahr*^−/−^ and *Ahr*^+/−^ mice- consistent with the PCR array-but there was no significant difference in the level of miR-135b induction between *Ahr*^−/−^ and *Ahr*^+/−^ mice (Fig. 6D). However, there was a significant difference in miR-96 expression between *Ahr*^−/−^ and *Ahr*^+/−^ mice exposed to cigarette smoke (Fig. 6E). Here, there was an approximately 4-fold increase in miR-96 after smoke exposure only in *Ahr*^−/−^ mice, but not AhR-expressing mice. When considered together, these data suggest that the AhR differentially controls the expression of miRNA in response to chronic cigarette smoke exposure.

### Kinetic profile of miR-96 reveals induction in *Ahr* ^−/−^ mice is associated with chronic- but not acute- cigarette smoke exposure

**Figure 7:**
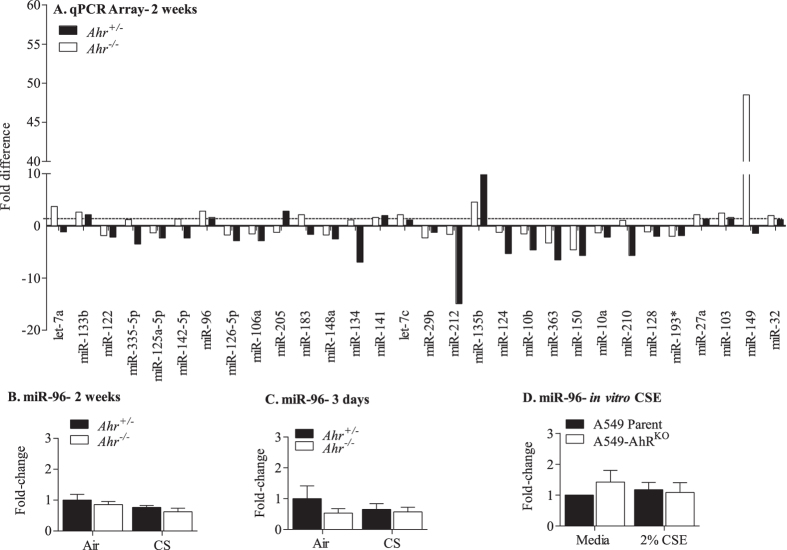
Acute and sub-chronic smoke exposure does not increase pulmonary miR-96 in *Ahr*^−/−^ mice. (**A**) qPCR Array- 2 weeks- miRNA analysis after 2 weeks of exposure to cigarette smoke indicated that there were fewer miRNA regulated by cigarette smoke. miR-96 was similar between *Ahr*^−/−^ and *Ahr*^+/−^ mice. (**B**) miR-96- 2 weeks- There was no significant difference in miR-96 after cigarette smoke exposure for 2 weeks. There was also no difference between *Ahr*^−/−^ and *Ahr*^+/−^ mice. (**C**) miR-96-3 days- There was no significant difference in miR-96 after exposure to CSE for 3 days. Results are presented as the mean ± SEM (n = 4–5 mice per group). (**D**) miR-96- *in vitro* CSE- There was no change in miR-96 in A549 cells with (A549 Parent) and without (A549-AhR^KO^) AhR expression. Results are presented as the mean ± SEM (n = 4 independent experiments).

Next, we wanted to know if the suppression of miR-96 by the AhR was reflective of chronic exposure, or if this regulation also occurred with more acute exposures. Using both our acute (3 days) and sub-chronic cigarette smoke exposure regimes (2 weeks), we have previously shown that AhR deficiency results in a heightened inflammatory response^10^,^22^ similar in nature to the one presented here (Figs 1 and 2). Using a qPCR array, we noticed that there was little change in miR-96 in either *Ahr*^−/−^ and *Ahr*^+/−^ mice after 2 weeks of cigarette smoke (Fig. 7A). In fact, there were remarkable fewer miRNAs changed at 2 weeks compared with 4 weeks of exposure (compared Figs 5 and 7). Validation of these array results confirmed that there was no significant change in miR-96 (Fig. 7B). There was also no change in miR-96 with 3 days of smoke exposure (Fig. 7C). Finally, we exposed A549 cells deficient in AhR expression (A549-AhR^KO^)^12^ to CSE, an *in vitro* surrogate of acute cigarette smoke exposure. There was also no change in miR-96 after exposure to CSE in either A549 Parent cells (which express the AhR) or A549-AhR^KO^ cells (Fig. 7D). Thus, we conclude that induction of pulmonary miR-96 in *Ahr*^−/−^ mice is reflective of a chronic cigarette smoke exposure regime.

### Mechanism of AhR-dependent regulation of miR-96 does not involve classic AhR activation

**Figure 8:**
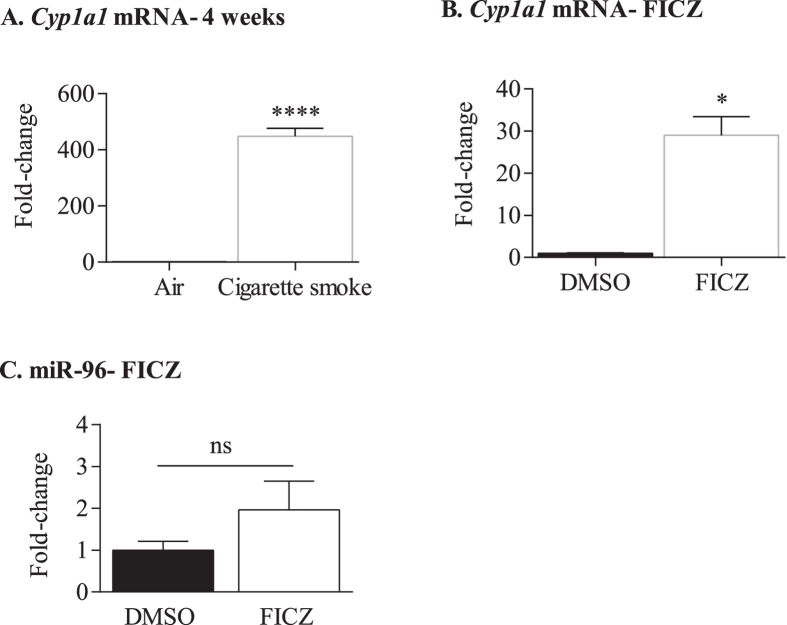
Regulation of miR-96 by the AhR does not require AhR activation. (**A**) *Cyp1a1* mRNA- 4 weeks- After 4 weeks of daily smoke exposure, there was a significant increase in pulmonary *Cyp1a1* mRNA. Results are presented as the mean ± SEM (n = 3 mice/group). (**B**) *Cyp1a1* mRNA- FICZ- There was a significant increase in *Cyp1a1* mRNA 6 hours after a single injection of FICZ. Results are presented as the mean ± SEM (n = 2 mice/group). (**C**) miR-96-FICZ- There was no significant induction (ns) in miR-96 upon exposure to FICZ compared to control (DMSO). Results are presented as the mean ± SEM (n = 4 mice/group).

Our data (Figs 5 and 6) show that AhR expression is important in suppressing miR-96 in response to chronic smoke exposure, which we predict is independent of classic AhR activation. Cigarette smoke contains AhR ligands- such as B[*a*]P- that induce this classic pathway (*i*.*e*. AhR translocation to the nucleus and the subsequent transcription of the AhR target gene *Cyp1a1*). We have previously shown that exposure to cigarette smoke for 2 weeks significantly increases pulmonary *Cyp1a1* mRNA only in *Ahr*^+/−^ mice^22^; now we show that this induction of *Cyp1a1* also occurs with chronic smoke exposure (Fig. 8A). When considered with the data in Fig. 6E, it suggests that classic AhR activation does not appreciably control miR-96 induction. Additional evidence to support this comes from experiments whereby we administered the high affinity endogenous AhR ligand FICZ^25^ to *Ahr*^+/−^ mice and evaluated pulmonary AhR activity. In these experiments, there was a significant increase in *Cyp1a1* mRNA in the lung (Fig. 8B), indicating strong AhR activation in the lung. However, there was no effect on miR-96 expression (Fig. 8C). We conclude that the suppression of miR-96 by the AhR occurs by a mechanism that is independent of classic AhR activation by either endogenous (FICZ) or exogenous (cigarette smoke) ligands.

### Pulmonary HuR and COX-2 expression after 4-week cigarette smoke exposure

**Figure 9:**
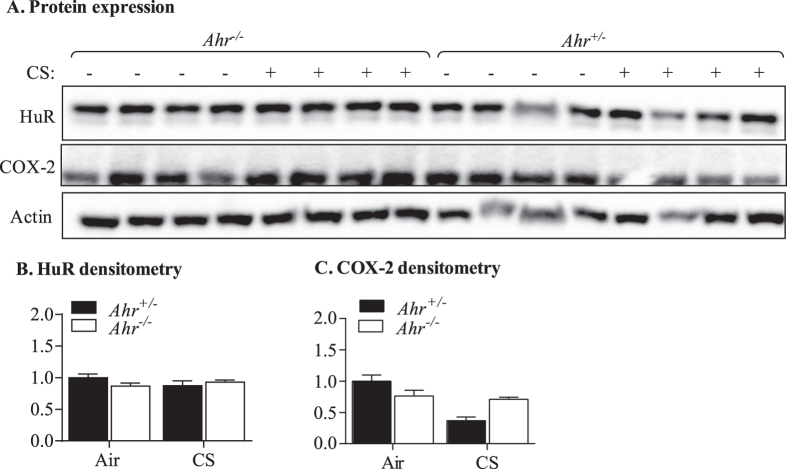
Expression of HuR and COX-2 in *Ahr*^+/−^ and *Ahr*^−/−^ mice after a 4-week cigarette smoke exposure. (**A**) Protein expression- Western blot shows HuR and COX-2 expression in whole lung homogenates from a 4-week cigarette smoke exposure regime. There is little perceptible difference in HuR or COX-2 expression between *Ahr*^+/−^ mice exposed to cigarette smoke and *Ahr*^−/−^ mice. (**B**) HuR densitometry- there was no significant difference in HuR expression. (**C**) COX-2 densitometry- There was less COX-2 expression *Ahr*^−/−^ mice exposed to CS for 4 weeks compared to *Ahr*^+/−^ mice. Antibodies were added sequentially to one membrane and images represent cropped blots for improved clarity. Results are presented as the mean ± SEM (n = 4 mice per group) and all samples were run on the same gel.

We have previously shown that the AhR controls the cellular localization of HuR in response to cigarette smoke^9^. HuR is a ubiquitous RNA-binding protein that is abundantly localized to the nucleus but shuttles between the nucleus and cytoplasm upon stimulation. It is believed that cytoplasmic localization is important for the mRNA-stabilizing effects of HuR, thereby increasing target protein expression, including those involved in inflammation and cancer progression^26^,^27^. Bioinformatic analysis (http://www.targetscan.org) indicated that HuR was a putative target of miR-96, leading us to speculate that AhR regulation of miR-96 may influence lung HuR expression. However, western blot analysis revealed that HuR is constitutively expressed in the lung, with no significant difference in total HuR expression between *Ahr*^−/−^ and *Ahr*^+/−^ mice exposed to cigarette smoke (Fig. 9A and B). One of the protein targets of both the AhR and HuR is COX-2^9^,^28^,^29^. There was a slight trend towards a change in COX-2 expression between cigarette smoke exposed *Ahr*^−/−^ and *Ahr*^+/−^ mice (Fig. 9A and C). Therefore, these results do not strongly support that HuR and COX-2 proteins are major targets for miR-96 in the lung.

### AhR-dependent regulation of pulmonary sulfiredoxin 1 (*Srxn1*) and FOXO3a by chronic cigarette smoke exposure

**Figure 10:**
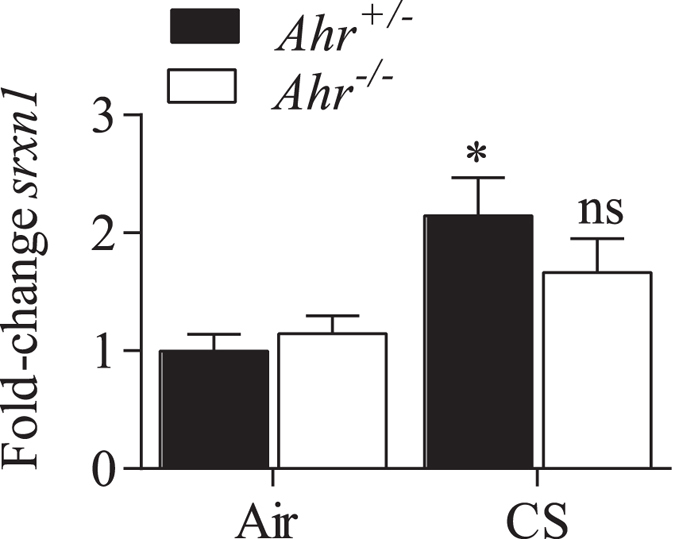
Pulmonary *srxn1* expression is increased by chronic cigarette smoke (CS) exposure in *Ahr*^+/−^ mice. There was a significant increase in *srxn1* mRNA expression in smoke-exposed *Ahr*^+/−^ mice (*p < 0.05 compared to air-exposed *Ahr*^+/−^ mice). There was no significant induction of *srxn1* in the lungs of *Ahr*^−/−^ mice (ns compared to air-exposed *Ahr*^−/−^ mice). Results are presented as the mean ± SEM (n = 4–5 mice per group).

**Figure 11:**
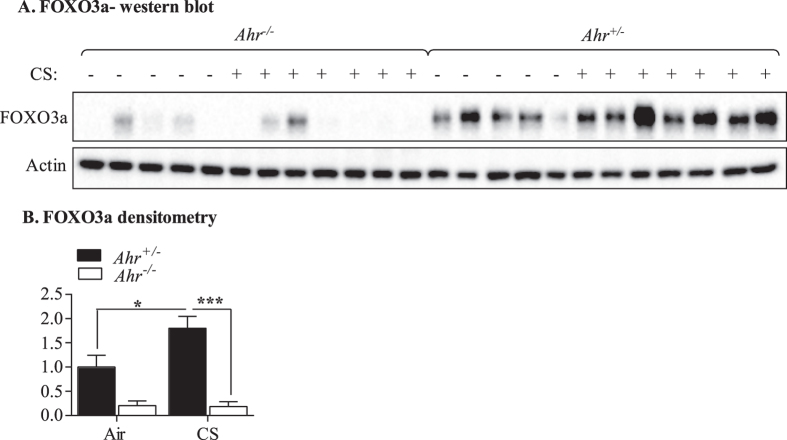
FOXO3a expression is increased by chronic cigarette smoke (CS) exposure in an AhR-dependent manner. (**A**) FOXO3a- western blot- There was a noticeable difference in FOXO3a expression between *Ahr*^−/−^ and *Ahr*^+/−^ mice, with FOXO3a being higher in the lungs of *Ahr*^+/−^ mice. (**B**) FOXO3a- densitometry- there was a significant increase in FOXO3a protein expression in cigarette smoke-exposed *Ahr*^+/−^ mice compared to air-exposed mice (*p < 0.05). This increase in FOXO3a in *Ahr*^+/−^ mice was significantly higher than smoke-exposed *Ahr*^−/−^ (***p < 0.001). Results are presented as the mean ± SEM (n = 5–7 mice per group) and all samples were run on the same gel.

We were the first to show *in vitro* that the AhR promotes the induction of Srxn1 expression by a mechanism that is independent of DRE binding^30^. Srxn1 is an endogenous antioxidant that protects against cigarette smoke-induced oxidative stress^31^. As miR-96 is also predicted to regulate Srxn1, we evaluated the expression of *Srxn1* in the lungs of *Ahr*^−/−^ and *Ahr*^+/−^ mice exposed to cigarette smoke for 4 weeks. There was a significant increase *Srxn1* mRNA levels only in cigarette smoke-exposed *Ahr*^+/−^ mice (Fig. 10); there was no induction of *Srxn1* in *Ahr*^−/−^ mice. This data support a role for the AhR in controlling *Srxn1*. Finally, we evaluated the expression of FOXO3a, another predicted target of miR-96, and a transcription factor that controls both inflammation and oxidative stress^19^,^20^. Our new data show that there is a significant induction in FOXO3a in the lungs of *Ahr*^+/−^ mice exposed to cigarette smoke for 4 weeks (Fig. 11). There was no induction in FOXO3a in *Ahr*^−/−^ mice, where the levels of FOXO3a remained significantly lower. Collectively our data suggest that miR-96 may contribute to non-canonical regulation of both *Srxn1* and FOXO3a by the AhR, and one mechanism by which AhR controls excessive inflammation caused by chronic smoke exposure.

## Discussion

Although much is known about the toxicological actions of the AhR in response to polycyclic aromatic hydrocarbons (PAHs) or polychlorinated dibenzo-p-dioxins (PCDDs), very little is understood about the physiological role of the AhR. However, we and others have shown that the potential biological function of the AhR extends well-beyond its transcriptional response to man-made toxicants; these studies have implicated the AhR in the regulation of development, immune homeostasis, cell death and inflammation^8^,^12^,^13^,^22^,^30^,^32^,^33^. Our new data presented herein suggest that the AhR is involved in the regulation of pulmonary miRNA, a group of ncRNA that are now recognized as major regulators of protein expression. Importantly, these data highlight role of the AhR in controlling miRNA levels in its quiescent state (*i*.*e*. in the absence of exogenous ligand), with there being a large number of miRNA expressed at lower levels in the lungs of *Ahr*^−/−^ mice compared to control mice. Many of the miRNA controlled by the AhR are known to be involved in endogenous regulator functions of the AhR- including proliferation and cell death (*e*.*g*. miR-137, miR-196a and miR-133b)^12^,^34^,^35^. These data suggest that the AhR is a homeostatic regulator of basal miRNA expression.

The AhR is highly expressed in the lung^36^, where it can encounter ligands present in cigarette smoke. Cigarette smoking is the foremost preventable cause of mortality worldwide, with an estimated 8–10 million deaths occurring per year by 2030^37^. Moreover, over one-half of all persistent smokers will die from a tobacco-related disorder, 80% of which is attributable to one of three diseases: cardiovascular disease (CVD), lung cancer, and chronic obstructive pulmonary disease (COPD)^38^,^39^. Cigarette smoke is a complex mixture, containing nearly 5000 chemicals, including carcinogens, reactive oxygen species and other chemicals, including AhR ligands such as B[*a*]P. We have shown *in vitro* and *in vivo* the AhR activating potential of cigarette smoke^9^,^13^,^22^. Here we show for the first time, that upon exposure to cigarette smoke, there was a dramatic increase in the number of miRNA in the lungs of both *Ahr*^−/−^ and *Ahr*^+/−^ mice. However, there were more miRNA upregulated in response to cigarette smoke in *Ahr*^−/−^ mice, suggesting that the AhR may also function to control miRNA levels and attenuate an over-abundant expression profile in response to inhalational exposures. Many of the miRNAs (*e*.*g*. miR-146a, miR-96) are expressed in the human lung, where their altered expression is strongly implicated in the pathogenesis of lung disease^24^,^40^,^41^. We then chose to validate the expression of five miRNA, including miR-196a, a miRNA involved in proliferation and apoptosis and one previously shown by us to be regulated by the AhR in lung fibroblasts^12^. There was no significant change in the levels of miR-196a in response to cigarette smoke in either *Ahr*^−/−^ and *Ahr*^+/−^ mice, consistent with our *in vitro* experiments where cigarette smoke extract (CSE) also did not increase miR-196a^12^. This is different compared to our results in the lung (Fig. 6), where the AhR did not appreciably control miR-196a levels. The reason for this difference is not clear, but suggests that there is some cell-specific regulation of miR-196a by the AhR (*i*.*e*. in lung fibroblasts but not other pulmonary cell types).

We were also surprised that there was little change in miR-146a expression in response to chronic exposure to cigarette smoke. miR-146a is a potent suppressor of inflammation^42^,^43^, including the suppression of cigarette smoke-induced COX-2 *in vitro*^23^. As such, miR-146a may play an important role in inflammation-associated diseases such COPD^24^,^44^, lung cancer^45^,^46^ and CVD^47^. There is evidence that miR-146a is transiently induced by inflammatory stimuli, including smoke exposure, where there is a rapid rise followed by a return to basal levels with 24 hours or so^40^,^48^. Thus, miR-146a may be initially increased by cigarette smoke (*i*.*e*. by initial exposure)- followed by a decline- such that a chronic exposure to cigarette smoke fails to sustain increased levels of miR-146a.

We also examined miR-135b expression, which was significantly upregulated by chronic exposure to cigarette smoke, with their being an approximately 20-fold increase in expression. Our data are not the first to report an increase in miR-135b. Recently, Halappanavar and colleagues demonstrated that miR-135b is significantly increased in the lungs of smoke-exposed mice as a mechanism to resolve the inflammatory response^49^, supporting an anti-inflammatory role for miR-135b. Although our current (Figs 1 and 2) and previous data^8^,^22^ show that the AhR is also a potent suppressor of smoke-induced pulmonary inflammation, these data do not support that miR-135b contributes significantly to the ability of the AhR to attenuate inflammation, given that there was no significant difference in miR-135b induction between *Ahr*^−/−^ and *Ahr*^+/−^ mice.

Of the five miRNAs from the array we chose to validate, we found that miR-96 expression was regulated by the AhR. Our data show that chronic cigarette smoke increased expression of miR-96 only in *Ahr*^−/−^ mice, suggesting that the AhR may in fact be acting as a repressor of miR-96 in response to inhaled toxicants. The role of miR-96 in cigarette smoke-induced disease is unknown, and we are the first to report on the induction of miR-96 by cigarette smoke. There is over-expression of miR-96 in chronic lung diseases where cigarette smoke is the primary environmental risk factor, including idiopathic pulmonary fibrosis (IPF) and non-small cell lung cancer (NSCLC)^50^,^51^. Therefore, our results on the induction in miR-96 could have implications for these diseases, as miR-96 has been shown to promote progression/invasion, cell proliferation and chemo-resistance in many types of cancers including prostate cancer^52^, colorectal cancer^53^ and NSCLC^54^. Although dioxin is classified as a human carcinogen- acting through the AhR- there is controversy for the role of the AhR in cancer development. In some studies, the AhR acts as a potent tumor suppressor, recently being shown to repress melanoma growth^55^, liver carcinogenesis^56^ and inflammation-associated colorectal tumorigenesis^57^. Genetic variations in *AHR* are also implicated in lung cancer susceptibility amongst smokers, where such variation may affect AhR protein expression and/or activity^58^,^59^,^60^. miR-96 is also significantly increased in lung cancer^50^, lending to the possibility that low AhR (due to genetic alteration) could enhance lung cancer susceptibility in smokers through increased expression of miR-96. In support of a role that AhR expression, but not AhR activation, regulates miR-96 is our data that both FICZ (an endogenous high affinity AhR ligand)^25^ and chronic cigarette smoke exposure increases *Cyp1a1* mRNA without altering miR-96 expression. Our findings are similar to reports that B[*a*]P induction of hepatic mRNA (>400 genes) occurred without changes in miRNA expression^61^. How the AhR suppresses miR-96 is not known but could be due to AhR-dependent interaction with pathways that normally suppress miR-96 levels, such as the Wnt/β-catenin pathway^62^.

The biological target(s) for miR-96 in our model has remained elusive. In response to chronic smoke exposure, the AhR maintains its ability to suppress pulmonary neutrophilia- similar to our published studies using acute and sub-exposure regimes^10^,^22^. Despite higher neutrophils in smoke-exposed *Ahr*^−/−^ mice, there was no significant change in the majority of the chemotactic cytokines we evaluated, with the exception of CCL20. However, a bioinformatics search failed to reveal direct inflammation-related mRNA targets for miR-96, including CCL20. During this investigation, *Srxn1* was identified as a putative target and our data show that there is less *Srxn1* in *Ahr*^−/−^ mice. *Srxn 1* is an anti-oxidant that protects against oxidative stress by restoring the activity of peroxiredoxins. There is a significant decrease in Srxn1 in COPD^31^, a chronic disorder caused primarily by cigarette smoke, and one whose pathogenesis is strongly associated with an oxidant/anti-oxidant imbalance. Given that we and others have shown that the AhR protects against oxidative stress^30^,^63^,^64^, it may be that *Srxn1* regulation by an AhR/miR-96 axis, in part, protects against the development of COPD.

The regulation of *Srxn1* by this AhR/miR-96 axis also links with another previously-identified target of miR-96- FOXO3a. FOXO3a is a member of the FOXO subfamily of forkhead-box transcription factors, and is one that suppresses inflammation and oxidative stress. miR-96 upregulation decreases FOXO3a^65^, and FOXO3a deficiency increases susceptibility to the development of COPD and lung cancer^66^,^67^,^68^. Now, we show that the AhR controls the expression of FOXO3a, whereby expression of FOXO3a is increased only in the lungs of *Ahr*^+/−^ mice exposed to cigarette smoke for 4 weeks. Given that FOXO3a inhibits NF-κB activity^20^, a transcription factor that promotes inflammation, lower FOXO3a levels could therefore contribute to the heightened smoke-induced inflammatory response caused by AhR deficiency. Thus, miR-96 upregulation may indirectly promote inflammation in *Ahr*^−/−^ mice via suppression of FOXO3a, rather than by directly targeting inflammatory genes. We do not anticipate that this AhR-miR-96-FOXO3a axis suppresses pulmonary inflammation from acute smoke exposure, as there was no change in miR-96 expression following acute and sub-chronic exposures *in vivo* or by utilizing CSE *in vitro*. While it has been shown that FOXO3a suppresses pulmonary inflammation in response cigarette smoke exposures- including neutrophilia^66^ - we consider it unlikely that FOXO3a is how the AhR suppress lung inflammation from acute smoke exposure. The lower FOXO3a in *Ahr*^−/−^ mice may also explain the lack of induction of Srxn1, as oxidative stress causes FOXO3a to translocate to the nucleus and bind to the promoter of target genes, including peroxiredoxin III (PrxIII)^69^, a component of the PRXIII/Srxn redox system. Additional studies are needed to elucidate the functional significance of increased miR-96 associated with AhR deficiency, and the potential role of this miRNA towards pulmonary disease development. In conclusion, this study establishes that the AhR is important in controlling the expression of pulmonary miRNA in response to chronic cigarette smoke exposure. Further exploration into the miRNAs and targets affected by the AhR is necessary to confirm biological importance of the AhR in these fundamental processes.

## Methods

### *In vivo* cigarette smoke exposure

AhR-knockout (*AhR*^−/−^; B6.129 Ahr^tm1^/J) C57BL/6 mice were obtained from Jackson Laboratory (Bar Harbor, ME) and bred in-house. This *Ahr*^−/−^ mouse strain carries a targeted deletion of exon 2. A breeding scheme of heterozygous *Ahr*^+/−^ to *Ahr*^−/−^ mice was used, rendering mice of the *Ahr*^+/−^ genotype as littermate controls. For cigarette smoke exposures, age-matched (range 10–14 weeks) and gender-matched *Ahr*^−/−^ and *Ahr*^+/−^ mice were exposed to cigarette smoke as described^22^. Briefly, research cigarettes (3R4F; University of Kentucky, Lexington, KY) were smoked according to the Federal Trade Commission protocol (1 puff/minute/cigarette of 2 s duration and 35-ml volume) in a SCIREQ inExpose Exposure System (SCIREQ, Montreal, QC). Mainstream cigarette smoke was diluted with filtered air and directed into the exposure chamber. Control and *Ahr*^−/−^ mice were exposed to cigarette smoke for 5 days a week for 4 weeks to mimic a chronic exposure scenario. The average TPM was 240 ± 8.2 mg/m^3^. Acute (3-day) and sub-chronic exposures (2-weeks) were also performed. Immediately after the final exposure, mice were euthanized by exsanguination. All animal procedures were approved by the McGill University Animal Care Committee (Protocol Number: 5933) and were carried out in accordance with the Canadian Council on Animal Care.

### Ligand administration *in vivo*

The AhR agonist 6-formylindolo(3,2-b)carbazole (FICZ) (Enzo Life Sciences Canada, Burlington, Ontario) was dissolved in DMSO and 1 μg mouse administered intraperitoneally (i.p); the lungs harvested 6 hours post-administration.

### Tissue Harvest and Bronchoalveolar Lavage (BAL) Collection

Following the last exposure, mice were anesthetized with Avertin (2,2,2-tribromoethanol, 250 mg/kg i.p.; Sigma-Aldrich, St. Louis, MO) and the mice euthanized by exsanguination. The lungs were excised and lavaged twice with 0.5 mL of PBS. After the bronchoalveolar lavage (BAL) fluid was centrifuged, the supernatant collected, the BAL cell pellets resuspended in PBS and the total cell number was determined by counting on a hemocytometer. Differential cell counts (at least 300 cells/sample) were performed after cytospin slide preparation (Thermo Shandon, Pittsburg, PA) and staining with Hema-Gurr Stain (Merck, Darmstadt, Germany). Lung tissue was collected and either frozen immediately in liquid nitrogen and stored at −80 °C for protein/western blot analysis or stored in RNAlater^®^ (Qiagen Inc., Valencia, CA).

### Detection of BAL Cytokine Levels

BAL fluid was collected as described above and stored at −80 °C until used. BAL cytokine levels were evaluated using Luminex^®^ technology (Milliplex xMAP, Millipore, Billerica, MA).

### Preparation of Cigarette Smoke Extract (CSE)

CSE was generated from research cigarettes (3R4F; Kentucky Tobacco Research Council (Lexington, KT)) as previously described^8^,^13^,^70^. An optical density of 0.65 (320 nm) was considered to represent 100% CSE^8^,^13^ and was diluted in serum-free MEM to 2% CSE^13^,^71^,^72^.

### Generation and culture of A549-AhR^ko^ cells

Generation of A549-AhR knockout (A549-AhR^ko^) cells was accomplished by zinc finger nucleases (ZFNs) as previously described^12^,^73^.

### miRNA PCR array

RNA was reverse transcribed and amplified using miScript II Reverse Transcription Kit and miScript miRNA PCR Array (Qiagen, Valencia, CA) according to the instructions provided. miRNA profiling for 96 murine miRNA (MAM-102A, Qiagen) was performed on whole lung homogenates or air- and smoke-exposed *Ahr*^+/−^ to *Ahr*^−/−^ mice; one representative sample per exposure condition/AhR genotype was randomly chosen on which to perform the array. miRNA expression was normalized to the housekeeping miRNA (Snord85, Snord66 and Snord68) and analyzed as fold-change relative to air-exposed heterozygous mice utilizing the web-based software system for the miRNA PCR array (Qiagen, http://pcrdataanalysis.sabiosciences.com/pcr/arrayanalysis.php).

### qRT-PCR for validation of miRNA expression

Lung tissue was homogenized in QIAzol Lysis Reagent, RNA purification was performed using the miRNeasy Micro Kit (QIAGEN). Resulting RNA samples were diluted accordingly to 10 ng/uL. miRNA expression was assessed by two-step TaqMan^®^ RT-PCR (Applied Biosystems, Carlsbad, CA) for miR-196, miR-146a, miR-135b, miR-96, miR-34c, and U6 snRNA, a small nuclear RNA (snRNA) used as an internal control for miRNA analysis. miRNA expression was normalized to the U6 snRNA levels.

### qRT-PCR

Total RNA was isolated from homogenized lung from *Ahr*^−/−^ and *Ahr*^+/−^ mice as described above. cDNA was generated from DNAase-treated RNA using iScript II Reverse Transcription Supermix (Bio-Rad, Mississauga, ON). Quantitative PCR was then performed by addition of 1 μl cDNA and 0.5 μM primers with SsoFast^TM^ EvaGreen^®^ (Bio-Rad). Data acquisition and analysis was performed on a CFX96 Touch^TM^ qPCR Detection System (Bio-Rad). Primers sequences are as previously published^22^,^30^. Melt curve analysis revealed the amplification of a single product, indicating specificity of the primer pairs. The fluorescence detection threshold was set above the non-template control background within the linear phases of PCR amplifications and the cycle threshold (Ct) of each reaction was detected. Gene expression was analyzed using the ΔΔCt method and results are presented as fold-change normalized to housekeeping gene (*β-actin*). Data are expressed as fold-change relative to the average level of air-exposed controls.

### Western blot

Total cellular protein from homogenized mouse lungs was prepared using RIPA buffer (Pierce; Thermo Scientific). Protein quantitation was performed with the bicinchoninic acid (BCA) method (Pierce, Rockford, IL). 20 μg of protein were fractionated on a 10% SDS-PAGE gel and electro-blotted onto Immun-blot PVDF membrane (Bio-Rad Laboratories, Hercules, CA). Antibodies against HuR (1:2000; Santa Cruz Biotechnology), COX-2 (1:1000) (Cayman Chemical, Ann Arbor, MI), FOXO3a (1:100) (Cell Signaling) and total Actin (1:50,000; Milipore, Temecula, CA) were used to assess changes in relative expression. Proteins were visualized using HRP-conjugated secondary antibodies (1:10,000) followed by enhanced chemiluminescence (ECL) and imaged using a ChemiDocTM XRS+ System (Bio-Rad).

### Statistical Analysis

GraphPad Prism v6.0 was used to perform all statistical analysis. All results are presented as the mean ± SEM. Statistically significant differences were assessed by two-way analysis of variance (ANOVA) on non-normalized values followed by a post-test for more than 2 groups or student’s t-test (groups of 2).

## Additional Information

**How to cite this article:** Rogers, S. *et al*. Aryl hydrocarbon receptor (AhR)-dependent regulation of pulmonary miRNA by chronic cigarette smoke exposure. *Sci. Rep.* **7**, 40539; doi: 10.1038/srep40539 (2017).

**Publisher's note:** Springer Nature remains neutral with regard to jurisdictional claims in published maps and institutional affiliations.
